# Diarrhea incidence in low- and middle-income countries in 1990 and 2010: a systematic review

**DOI:** 10.1186/1471-2458-12-220

**Published:** 2012-03-21

**Authors:** Christa L Fischer Walker, Jamie Perin, Martin J Aryee, Cynthia Boschi-Pinto, Robert E Black

**Affiliations:** 1 Johns Hopkins Bloomberg School of Public Health, Department of International Health, 615 N. Wolfe St, Baltimore, MD 21205, USA; 2 Division of Biostatistics, Department of Oncology, Sidney Kimmel Comprehensive Cancer Center, Johns Hopkins University, Baltimore, Maryland 21205, USA; 3 Department of Child and Adolescent Health and Development, World Health Organization, Avenue Avia 20, Geneva 27, Switzerland

## Abstract

**Background:**

Diarrhea is recognized as a leading cause of morbidity and mortality among children under 5 years of age in low- and middle-income countries yet updated estimates of diarrhea incidence by age for these countries are greatly needed. We conducted a systematic literature review to identify cohort studies that sought to quantify diarrhea incidence among any age group of children 0-59 mo of age.

**Methods:**

We used the Expectation-Maximization algorithm as a part of a two-stage regression model to handle diverse age data and overall incidence rate variation by study to generate country specific incidence rates for low- and middle-income countries for 1990 and 2010. We then calculated regional incidence rates and uncertainty ranges using the bootstrap method, and estimated the total number of episodes for children 0-59 mo of age in 1990 and 2010.

**Results:**

We estimate that incidence has declined from 3.4 episodes/child year in 1990 to 2.9 episodes/child year in 2010. As was the case previously, incidence rates are highest among infants 6-11 mo of age; 4.5 episodes/child year in 2010. Among these 139 countries there were nearly 1.9 billion episodes of childhood diarrhea in 1990 and nearly 1.7 billion episodes in 2010.

**Conclusions:**

Although our results indicate that diarrhea incidence rates may be declining slightly, the total burden on the health of each child due to multiple episodes per year is tremendous and additional funds are needed to improve both prevention and treatment practices in low- and middle-income countries.

## Background

Diarrhea remains a leading cause of mortality among children under 5 years of age around the world [1]. The burden of Diarrheal disease disproportionately affects young children in low- and middle-income countries who have higher incidence rates due to inadequate water and sanitation and nutritional risk factors, such as suboptimal breastfeeding and zinc and vitamin A deficiency [2-4]. Children living in impoverished areas also have higher case-fatality rates compared to children living in high-income countries due to lack of access to quality health care and timely and effective treatment with oral rehydration solution (ORS) and zinc [5].

There is currently no widely available source for diarrhea incidence estimates. While cross-sectional surveys such as Demographic and Health Surveys routinely gather 2-week point prevalence rates for low- and middle-income countries around the world, these data cannot be used to generate incidence rates because they can be highly affected by seasonal variation in incidence. Age-specific incidence rates acquired from cohort studies including at least 1 year of surveillance are the best source of data, but these are not available for every country. Therefore, we rely on modeling to generate estimates by country and region, and for the world.

Snyder and Merson first estimated diarrhea incidence for young children to be 2.2 episodes/year in 1980 using available data published between 1954 and 1979 [6]. Ten years later Bern et al. published an update of this review using similar methodology, including more recent studies, and estimated children to have 2.6 episodes of diarrhea/year [7]. In 2003, Kosek et al. provided another updated estimate of diarrhea morbidity concluding that children have 3.2 episodes of diarrhea/year [8]. Given it has been nearly 10 years since the last published estimate of diarrhea morbidity, there is a great need for updated estimates of incidence rates for calculating burden of disease and for planning at the country level. In addition, numerous studies have been published since the last review; thus, we sought to include updated data, as well as improve upon past search strategies to expand the contributing of literature.

## Methods

### Systematic review and data abstraction

**Figure 1:**
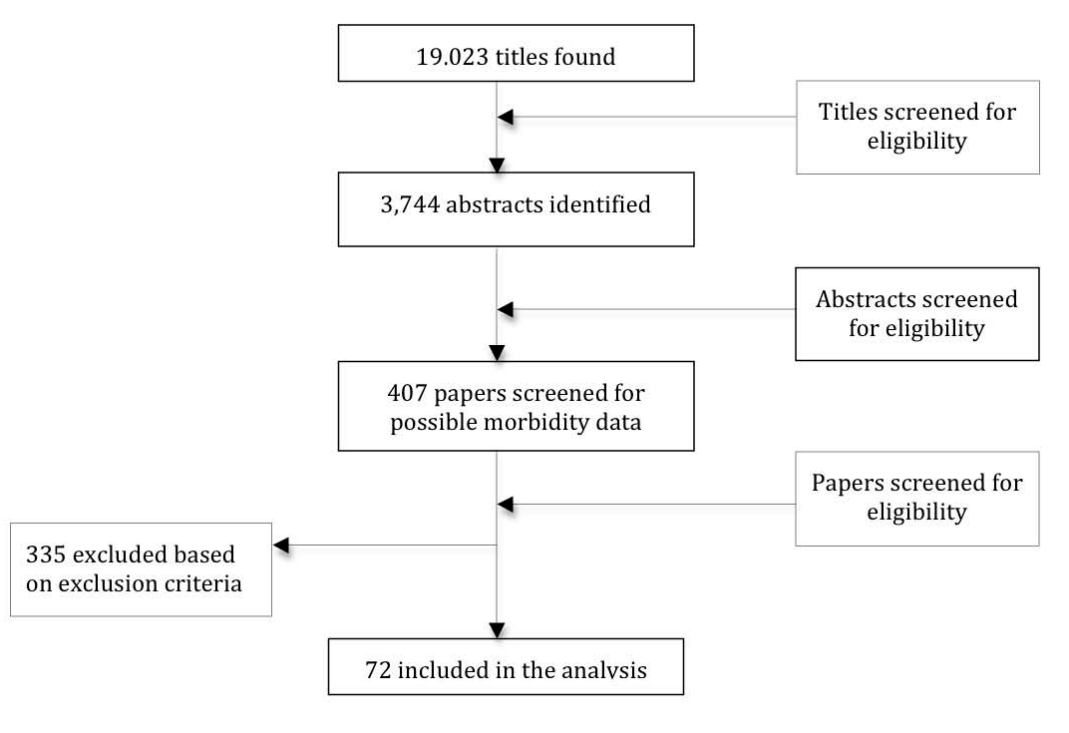
**Flow chart diagram of the systematic review process**.

We conducted a systematic literature review to identify community-based cohort studies of children 0-59 mo of age with at least 12 mo of diarrhea surveillance. Diarrhea was defined as 3 or more loose or watery stools in 24 h. We searched PubMed, Embase, Global Health, and the WHO Regional Databases and used all combinations of the following search terms: *diarrh(o)ea, child, morbidity, incidence, surveillance, burden of disease*, and *prevalence*. We sought papers with diarrhea incidence data. We included papers in English, French, Spanish, and Italian published between 1980 and Aug 31, 2010 in our search. We excluded papers with recall periods beyond 2 weeks and those conducted in unrepresentative populations (i.e. low birth weight infants or children with chronic disease). We sought unpublished datasets by contacting authors known globally for their large community-based study sites, but held them to the same inclusion/exclusion standards. The initial search was conducted in June 2008 and was updated on Sept 15, 2010. We first screened titles and abstracts, and then full manuscripts for all potentially eligible papers (Figure 1). Final papers were abstracted by two trained data abstractors into a standardized abstraction spreadsheet.

### Data analysis

We used empirical data from the literature on diarrhea incidence to estimate age-specific incidence rates for 139 low- and middle-income countries, ^a ^based on each country's WHO region and under five mortality rate. Reported incidence rates varied widely in the literature and many studies presented truncated age cohorts (for example, a study may include only children 0-23 mo of age) or non-standard age groupings (for example, the incidence for children 8-21 mo of age in lieu of 6-11 and 12-23 mo of age). Standard age categories include 0-5 mo, 6-11 mo, 12-23 mo, 24-35 mo, 36-47 mo, and 48-59 mo. If all studies reported incidence rates using standard age groups, a simple regression model could be used to estimate the effect of covariates (e.g. WHO region and under five mortality) on age-specific incidence. However, because a range of non-standard age groups are reported and age-group specific data is sparse in many regions, we made the simplifying assumption that the shape of the incidence rate age curve is the same across all countries. (e.g. the ratio of 12-23 mo incidence is a fixed multiple of the 6-11 mo incidence regardless of country). Country-specific characteristics (i.e. WHO region and under five mortality) shift the incidence curve up or down, but do not alter the shape of the curve. The specific shape of this curve is estimated by all studies which included 0-59 and at least 1 age category; for this analysis this included 65 of 72 studies.

To convert non-standard age group reports into preset age groups we modeled through a two-stage iterative process using the Expectation-Maximization (EM) algorithm. The EM algorithm can be applied to data sets with missing or incomplete data [9-11]. The analysis is separated into two distinct steps. We first used the current estimate of the incidence rate age curve to convert the observed age groups into the standard age groups. For example, an incidence rate reported for the age group 1-23 months would be converted into estimates for 0-5, 6-11, and 12-23 mo. This step is similar to an imputation where we obtain best guess estimates of the incidence rates that would have been observed had the standard age groups been reported. In the second step we then fit a regression model to the standardized age group incidence rates to estimate the parameters for curve shape, WHO region and under five mortality rate. The updated curve shape parameters are then used again in the first step and the process iterates repeatedly until the incidence estimates are stable.

We weighted country level incidences predicted by this model by the population of children under the age of five to calculate regional age-specific estimates. In order to approximate uncertainty for regional rates, we used the nonparametric bootstrap [12] to resample study data by WHO region. We fit the model repeatedly with resampled data and weighted averages taken from the incidences predicted by country. We report the 2.5th and 97.5th percentiles from the resulting distribution of incidence for each region using a standard nonparametric bootstrap estimator that has an approximate 0.95 probability of including the true regional incidence represented by the study data [13]. We also used these distributions of predicted incidences by region to determine if observed differences were spuriously detected and within random prediction error, or whether the difference in incidence over time is statistically supported by data. Because incidence is modeled using only WHO region and under five mortality rates, the estimated change in incidence over time is driven by changes in under five mortality. Under 5 mortality steadily declined from 1990 to 2010 in most countries so testing for a decline in incidence is equivalent to testing that this relationship is statistically significant.

We then calculated the total number of episodes of diarrhea among the 139 low- and middle-income countries included in this analysis by multiplying our estimated regional diarrhea incidence rates for all children 0-59 mo of age by the total population of children under 5 years of age for both 1990 and 2010.

## Results

### Systematic literature review

**Figure 2:**
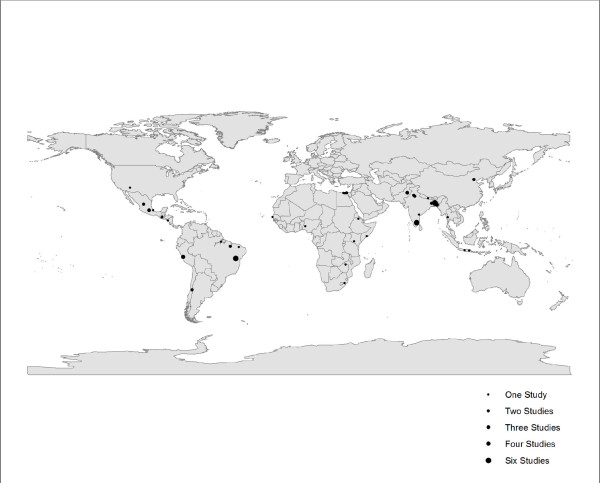
**Location of studies reporting Diarrhea incidence outcomes for children and adults**.

We screened more than 19,000 possible titles to identify 72 studies that met all inclusion and exclusion criteria (Figure 1). The studies were from 5 of the 6 WHO regions (all except Europe) (Figure 2) and were published between 1980 and 2010 representing data collection between 1976 and 2006. We abstracted age-specific incidence rates for all age categories described in the studies; this included 50 different age categories and 187 individual incidence rates from among these 72 studies that were included in the age adjustment and modeling methods. Study characteristics including site, study type, urban or rural setting, year of data collection, sample size, and incidence rate by age for the 72 included studies can be found in Additional file 1.

### Diarrhea Incidence by age and region

Using the age-specific data we produced country-specifics estimates for each age category (0-5 mo, 6-11 mo, 12-23 mo, and 24-59 mo) for 1990 and 2010 (Additional file 2). The country level data were then aggregated up to create regional estimates for 1990 and 2010 (Tables 1 and 2). For 1990 diarrhea incidence rates were highest among children 6-11 mo of age (5.3 episodes/child year) and lowest among children 24-59 mo of age (2.7 episodes/child year. Overall children experienced 3.4 episodes of diarrhea per year. Using 1990 population estimates for children 0-59 mo of age we estimate that there were nearly 1.9 billion episodes of diarrhea among children living in the 139 countries included in these analyses (Table 3). For 2010 diarrhea incidence rates remained highest among infants 6-11 mo of age and dropped from 5.3 to 4.5 episodes per year during this 20-year period. Again, children 24-59 mo of age had the lowest estimated incidence rate at 2.3 episodes/child year. Overall incidence rates declined from 1990 to 2010 (*p *< 0.05) in all regions with the greatest decline observed in Africa (4.2 to 3.3 episodes/child year). In 2010, each child experienced an estimated 2.9 episodes resulting in nearly 1.7 billion diarrhea episodes among children less than 5 years of age in low- and middle-income countries.

**Table 1:** Diarrhea incidence rates by WHO region for 1990

	Age				
	**Episodes of Diarrhea per child year (Uncertainty bounds)**				
Region	0-5 months	6-11 months	12-23 months	24-59 months	0-59 months
Africa	4.3 (2.9, 7.6)	6.5 (4.7, 11.6)	5.3 (3.6, 9.3)	3.4 (2.3, 6.0)	4.2 (3.0, 7.3)
Americas	4.6 (3.4, 5.8)	7.0 (5.3, 8.4)	5.7 (4.3, 6.6)	3.6 (2.6, 4.6)	4.5 (3.4, 5.3)
Eastern Mediterranean	3.6 (1.8, 5.9)	5.4 (3.0, 8.3)	4.4 (2.4, 6.6)	2.8 (1.6, 4.3)	3.5 (2.0, 5.2)
Europe	4.5 (3.3, 5.7)	6.9 (5.2, 8.2)	5.6 (4.3, 6.5)	3.6 (2.5, 4.5)	4.4 (3.4, 5.2)
South East Asian	3.1 (2.0, 4.6)	4.7 (3.3, 6.3)	3.8 (2.7, 5.0)	2.4 (1.8, 3.1)	3.0 (2.2, 3.8)
Western Pacific	2.6 (1.5, 3.3)	4.0 (2.2, 4.8)	3.2 (1.8, 3.7)	2.0 (1.2, 2.4)	2.5 (1.4, 2.8)
Global	3.4 (2.6, 4.7)	5.3 (4.4, 6.4)	4.3 (3.5, 5.1)	2.7 (2.2, 3.3)	3.4 (2.9, 3.9)

**Table 2:** Diarrhea incidence rates by WHO region for 2010

	Age				
	**Episodes of Diarrhea per child year (Uncertainty bounds)**				
Region	0-5 months	6-11 months	12-23 months	24-59 months	0-59 months
Africa	3.4 (2.1, 5.6)	5.1 (3.4, 8.1)	4.2 (2.7, 6.4)	2.7 (1.7, 4.2)	3.3 (2.2, 5.1)
Americas	4.1 (2.9, 5.2)	6.2 (4.7, 7.5)	5.0 (3.9, 5.8)	3.2 (2.3, 4.0)	4.0 (3.1, 4.7)
Eastern Mediterranean	3.1 (1.5, 5.2)	4.7 (2.4, 7.0)	3.8 (1.9, 5.4)	2.4 (1.3, 3.6)	3.0 (1.6, 4.4)
Europe	4.1 (3.0, 5.3)	6.3 (4.8, 7.6)	5.1 (3.9, 5.9)	3.2 (2.3, 4.1)	4.0 (3.1, 4.7)
South East Asian	2.4 (1.4, 4.0)	3.7 (2.2, 5.6)	3.0 (1.8, 4.3)	1.9 (1.2, 2.6)	2.4 (1.5, 3.3)
Western Pacific	2.3 (1.3, 3.1)	3.5 (2.0, 4.4)	2.9 (1.6, 3.3)	1.8 (1.1, 2.2)	2.3 (1.3, 2.6)
Global	2.9 (2.1, 4.3)	4.5 (3.4, 5.7)	3.6 (2.8, 4.4)	2.3 (1.8, 2.8)	2.9 (2.3, 3.4)

**Table 3:** Total Diarrhea episodes among children under 5 years of age living in low- and middle-income countries for 1990 and 2010

	1990			2010		
WHO Region (number of low-/middle income countries included in analysis)	Episodes/child year	Population of children under 5 years	Total	Episodes/child year	Population of children under 5 years	Total
Africa (45)	4.2	92,112,710	386,873,382	3.3	135,986,600	448,755,780
Americas (29)	4.5	55,097,140	247,937,130	4.0	55,069,550	220,278,200
Eastern Mediterranean (15)	3.5	60,682,760	212,389,660	3.0	66,458,970	199,376,910
Europe (23)	4.4	37,236,710	163,841,524	4.0	27,836,060	111,344,240
South East Asian (11)	3.0	175,764,800	527,294,400	2.4	182,015,100	436,836,240
Western Pacific (16)	2.5	143,774,300	359,435,750	2.3	110,854,400	254,965,120
**Total Diarrhea Episodes**			1,897,771,846			1,671,556,490

## Discussion

We estimated diarrhea incidence rates among children under 5 years of age living in low- and middle-income countries in 6 WHO regions and for the world for 1990 and 2010. We found that while diarrhea incidence has declined from an estimated 3.4 episodes/child year in 1990 to 2.9 episodes/child year in 2010, the highest burden of disease has remained consistent with respect to age (i.e. with 6-11 mo olds having the highest incidence). The decline we present here from 1990 and 2010 is strongly correlated with the overall decline in under 5 mortality rates observed during this same period. With the model presented here we observe overlapping uncertainty bounds for the point estimates, yet still observe a statistically significant decline over time when testing the statistical significance of the rate of decline using the bootstrap model.

Our estimates are in line with previously published estimates of 2.2 episodes/child year in 1980, [6] 2.6 episodes/child year in 1990, [7] and 3.2 episodes/child year in 2003 [8]. Differences can be easily explained by our updated search strategy and difference in age-adjustment as part of the analytic methods. We conducted an extensive literature review and included languages and databases that may have been overlooked in the past. In addition, the passage of time afforded us the opportunity to include more studies that were not included in the past. With these changes the total number of included studies went from 27 in the most recent 2003 estimate [8] to 72 in our current review and analysis. However, despite this increase in total studies identified, very few (n = 14) of theses are from 2000 or beyond thus the need for more recent data to better estimate current incidence rates remains an issue.

We have also employed a number of analytic changes that we believe strengthen the estimation process and the certainty of our final estimates. We used an analytic method not previously used in diarrhea incidence calculations, i.e. the Expectation Maximization algorithm, to incorporate incidence rates reported by authors for non-standard age categories (i.e. any age category other than 0-5, 6-11, 12-23, 24-35, 36-48, and 0-59 mo). In the past, age-specific data was only used if it fit into these (or fewer) standard age categories. Unfortunately this limited the inclusion of much of the age-specific data. We found data in 50 different unique age categories. Because incidence rates vary by age, the inclusion of as much age-specific data as possible is critical to the improvement of the estimation process. We also used country level covariates as part of the regression model. These country-level data improve upon taking simple weighted means or median incidence rates by allowing for the influence to vary based on the location of the input studies and national level data.

We recognize our study has several limitations. While the 72 studies identified and included in this analysis represent a substantial improvement upon past estimates, this number of studies is still low considering an inclusion period for studies of more than 30 years. In addition, while we are estimating using all data for two time periods it should be pointed out that only 14 of these studies contained data from the year 2000 and beyond. Thus, estimates for the recent past (i.e. 2010) are predominantly based on older data relying on changes in model level covariates to adjust for diarrhea incidence changes over time rather than empirical data. This lack of data highlights the paucity of routine cohort studies conducted within the last decade raises great concern given that the estimates presented here continue to suggest that diarrhea is an enormous health burden for children in low- and middle-income countries.

We modeled diarrhea incidence for each country using published country level and under 5 mortality rates. Ideally a model would include covariates such as water and sanitation, breastfeeding practices, rotavirus vaccine coverage, etc., i.e. indicators that are widely known to directly influence diarrhea morbidity rates. We attempted to include these indicators and sought study level data for each of these during the initial abstraction process; however, unfortunately most published papers did not include study level data on these covariates. In our previous models, where study level data were missing, we used country level indicators from DHS surveys, but during the modeling process these covariates did not prove to be predictive of diarrhea incidence and fell out of all early versions of the regression models. This is not surprising because small study sites can often be drastically different from the country where they are conducted with regard to common socioeconomic and child health indicators collected in large cross sectional surveys. In addition, some interventions such as vitamin A supplementation, are better linked to reductions in diarrhea mortality than to reduction in diarrhea incidence. In our final model we included only under 5 mortality rates. While we recognize that this indicator could also be improved with local, site-specific data to better relate the study level incidence, it proved to be more statistically sound than indicators such as access to water and sanitation, that were tested in early versions. Gross National Income (GNI) also fell out of the model due to colinearity with reduction in under 5 mortality rates over time.

Introducing new analytic methods has the potential for new sources of error. We chose to use the ratio of age specific rates as a feature of the incidence data. These ratios can be observed in the raw study data where at least two ages are reported exactly as we have specified, i.e., as 0-5 mo, 6-11 mo, 12-23 mo, or 24-59 mo. Thirteen studies reported incidence rates for 0-5 mo and 6-11 mo; the average ratio of incidence (6-11 mo compared to 0-5 mo) in the study data was 1.392, while our model predicts a ratio of 1.389. For the age groups 6-11 mo and 12-23 mo (n = 10 studies), the average ratio of incidence among study data for 12-23 mo compared to that for 6-11 mo was 0.780, while our model predicts a ratio of 0.779. There are no studies where 24-59 mo is reported along with another standard age group.

Diarrhea incidence remains a tremendous burden on children in low- and middle-income countries. Diarrhea has been shown to have a lasting influence on nutritional status in that an increase in diarrhea prevalence has been linked to an increased risk of stunting [14] and diarrhea has been shown to be a risk factor for pneumonia [15]. As diarrhea mortality rates continue to decline it becomes increasingly important to emphasize the role of frequent diarrhea episodes experienced by the surviving children. While both case fatality rates and incidence rates may be declining, both declines are occurring too slowly given the breadth of available therapeutic and preventive interventions. The coverage of therapeutic interventions, namely ORS and zinc remains poor throughout developing countries. Although water and sanitation services have improved since 1990, other possible modes of enteric pathogen transmission, such as contaminated food or poor personal hygiene, may not have. Exclusive breastfeeding practices for the first 6 mo of life and continued breastfeeding until 24 mo of age continue to need improvement in nearly all low- and middle-income countries. Because diarrhea has several transmission routes, it can be hypothesized that simply removing one such route does not eliminate the risk of diarrhea [16]. It has been observed that the protective effect of improved water quality is greater among communities with improved sanitation conditions compared to higher contaminated areas [17]. Thus, for substantial changes in diarrhea incidence rates due to the most common pathogens, multiple transmission routes need to be eliminated simultaneously at the household and community level.

## Conclusions

Children in low- and middle-income countries remain at risk for frequent diarrhea episodes and thus for secondary infections and the long-term sequel of delayed, or never attained growth. It is becoming increasingly important to focus on improved delivery strategies to enhance access to diarrhea prevention and treatment programs as well as the development of new technologies including vaccines which may help future generations.

## Competing interests

The authors declare that they have no competing interests.

## Authors' contributions

CLFW designed the study, conducted the search, interpreted the final results, and wrote the manuscript. JP and MA conducted the analysis and assisted with the manuscript. CPB and REB also helped to design the study, conduct the search, interpret the results, and contributed to the final manuscript. All authors read and approved the final manuscript.

## Funding

The study was supported by grants from the Bill and Melinda Gates Foundation to the US Fund for UNICEF for the Child Health Epidemiology Reference Group of the World Health Organization and UNICEF and to the University of Washington for the Global Burden of Disease Project. No funding bodies played any role in the design, writing or decision to publish this manuscript.

## Endnote

^a ^Africa: Algeria, Angoloa, Benin, Botswana, Burkina Faso, Burundi, Cote d'Ivoire, Cameroon, Cape Verde, Centra African Republic, Chad, Comoros, Congo, Democratic Republic of Congo, Eritrea, Ethiopia, Gabon, Gambia, Ghana, Guinea-Bissau, Guinea, Kenya, Lesotho, Liberia, Madagascar, Malawi, Mali, Mauritania, Mauritius, Mozambique, Namibia, Niger, Nigeria, Rwanda, Sao Tome and Principe, Senegal, Seychelles, Sierra Leone, South Africa, Swaziland, Togo, Uganda, United Republic of Tanzania, Zambia, Zimbabwe. Americas: Antigua and Barbuda, Argentina, Belize, Bolivia, Brazil, Chile, Colombia, Costa Rica, Cuba, Dominica, Dominican Republic, Ecuador, El Salvador, Grenada, Buatemala, Guyana, Haiti, Honduras, Jamaica, Mexico, Nicaragua, Panama, Peru, Saint Lucia, Saint Vincent, Suriname, Uruguay, Venezuala. Eastern Mediterrean: Afghanistan, Djibouti, Egypt, Iran, Iraq, Jordan, Lebaon, Libyan Arab Jamahiriya, Moocco, Pakistan, Somalia, Sudan, Syrian Arab Republic, Tunisia, Yemen. Europe: Albania, Armenia, Azerbaijan, Belarus, Bosnia and Herzegovinia, Bulgaria, Georgia, Kazakhstan, Kyrgyzstan, Latvia, Lithuania, Moldova, Montenegro, Occupied Palestian Territory, Romania, Russian Federation, Serbia, Tajikistan, TFYR Macedonia, Turkey, Turkmenistan, Ukraine, Uzbekistan. South East Asia: Bangaldesh, Bhutan, Dem. People's Republic of Korea, India, Indonesia, Maldives, Myanman, Nepal, Sri Lanka, Thailand, Timore Leste. Western Pacific: Cambodia, China, Fiji, Kiribati, Lao People's Democratic Republic, Malaysia, Marshall Islands, Mongolia, Papua New Guinea, Philippines, Samoa, Solomon Islands, Taiwan, Tonga, Vanuatu, Viet Nam.

## Pre-publication history

The pre-publication history for this paper can be accessed here:

http://www.biomedcentral.com/1471-2458/12/220/prepub

## Supplementary Material

Additional file 1 **Characteristics of included studies**. Click here for file

Additional file 2 **Diarrhea Incidence Rates for Included Countries**. Click here for file
